# Combined Action of Piperitenone Epoxide and Antibiotics Against Clinical Isolates of *Staphylococcus aureus* and *Escherichia coli*

**DOI:** 10.3389/fmicb.2019.02607

**Published:** 2019-11-19

**Authors:** Athanasios Alexopoulos, Athanasios C. Kimbaris, Stavros Plessas, Ioanna Mantzourani, Chrysa Voidarou, Olga Pagonopoulou, Christina Tsigalou, Maria Fournomiti, Christos Bontsidis, Elisavet Stavropoulou, Virginia Papaemmanouil, Eugenia Bezirtzoglou

**Affiliations:** ^1^Laboratory of Microbiology, Biotechnology and Hygiene, Faculty of Agricultural Development, Democritus University of Thrace, Orestiada, Greece; ^2^Laboratory of Chemistry & Biochemistry, Faculty of Agricultural Development, Democritus University of Thrace, Orestiada, Greece; ^3^Department of Agricultural Technology, Faculty of Agricultural Technology, Food Technology and Nutrition, Technological Educational Institute of Epirus, Arta, Greece; ^4^Laboratory of Physiology, School of Medicine, Democritus University of Thrace, Alexandroupolis, Greece; ^5^Laboratory of Microbiology, Medical School, Democritus University of Thrace, Alexandroupolis, Greece; ^6^Service de Médecine Interne, Centre Hospitalier Universitaire Vaudois, Lausanne, Switzerland; ^7^Microbiological Laboratory, “Metaxa” Anticancer Hospital, Piraeus, Greece

**Keywords:** piperitenone epoxide, antibiotics, *Escherichia coli*, *Staphylococcus aureus*, minimum inhibitory concentration, fractional inhibitory concentration, fractional inhibitory concentration index

## Abstract

The aim of this study was to determine the antimicrobial efficiency of piperitenone epoxide (PEO) – a principal component of various aromatic plants’ essential oil – in combination with various antibiotics against 28 strains of *Staphylococcus aureus* and 10 strains of *Escherichia coli* isolated from clinical samples. *Mentha spicata’s* essential oil, initially collected by hydrodistillation, was then subjected to flush column chromatography affording PEO of high purity. Minimum inhibitory concentrations of PEO alone and in combination with various concentrations of antibiotics were assessed using the microdilution method. The combined action was estimated calculating the fractional inhibitory concentration (FIC) index from checkerboard assays. Our results showed that the average minimum inhibitory concentration (mg/l) of PEO alone against *E. coli* was 512 ± 364.7 μg/ml, which was significantly higher than 172.8 ± 180.7 μg/ml observed for *S. aureus*. From checkerboard assays, FIC values below the 0.5 index, indicating synergy, were observed for 59% of the drugs tested. Twelve percent of FIC index values were between 0.5 and 1, indicating additive effects, while 21% were indifferent. According to our results, PEO could be a promising antimicrobial compound when combined with specific antibiotics and deserves further study.

## Introduction

The increase in antibiotic resistant strains is a global public health concern in the continuous fight against pathogens. Owing to the lack of efficient drugs, it is estimated that 400,000 infections and more than 25,000 deaths occur annually in the European Union alone (ECDC/EMEA, 2009; Bush et al., 2011), creating also a significant economic impact of over €1.5 billion (Smith and Coast, 2013; Prestinaci et al., 2015). As alternatives to antibiotics, many researchers have explored the use of essential oils (EOs) or other bioactive compounds occurring naturally as secondary metabolites of aromatic and medicinal plants (Hammer et al., 1999; Lambert et al., 2001; Bakkali et al., 2008; Alexopoulos et al., 2011; Fournomiti et al., 2015; Saviuc et al., 2015). EOs are oily liquids rich in aromatic compounds, which are extracted from plant material mainly by steam- or hydrodistillation (Asbahani et al., 2015). Owing to their antiseptic, antibacterial, antifungal, anti-inflammatory, antinociceptive, anticancer, antioxidant, and analgesic properties (Adorjan and Buchbauer, 2010), they have a long history of use in traditional medicine (Raut and Karuppayil, 2014), cosmetology (Aburjai and Natsheh, 2003), crop protection (Dayan et al., 2009), and also in food preparation and preservation (Burt, 2004). The mechanism of antibacterial action of EOs is attributed either or collectively to the disruption of the bacterial cytoplasmic membrane and various diverse effects upon cellular metabolism (Lambert et al., 2001; Ultee et al., 2002; Di Pasqua et al., 2007).

However, despite their traditional use and proven biological properties, the use of EOs as antibacterial agents is constrained from limitations or disadvantages in their efficiency, variation in composition, toxicity, usability, bacterial resistance, and lack of knowledge of their mode of action (Bakkali et al., 2008; Baser and Buchbauer, 2015; Owen and Laird, 2018). In this context, as an alternative strategy, the combined action of existing antibiotics with complex phytochemicals or with their individual components was suggested from various investigators (Hemaiswarya et al., 2008; Wagner and Ulrich-Merzenich, 2009; Hyldgaard et al., 2012; Langeveld et al., 2014; Magi et al., 2015; Owen and Laird, 2018). This strategy combines the multidrug resistant modifier action of phytochemicals with the selectivity of antibiotics to overcome the intrinsic or acquired resistance mechanisms of bacteria with promising results toward their efficacy and commercialization viability (Hemaiswarya et al., 2008; Owen and Laird, 2018).

In 2001, Hu et al. (2001) successfully inhibited β-lactamase in β-lactamase-producing *Staphylococcus aureus* combining epigallocatechin-gallate and ampicillin/sulbactam, while Sakagami et al. (2005) inhibited methicillin-resistant *S. aureus* and vancomycin enterococci via the combination of α-mangostin with vancomycin. Since then, various studies have proven the successfulness of such combinations, with some of them demonstrating a remarkable up to 256-fold reduction in the antibiotic concentration (Owen and Laird, 2018). Similar results from the combined use of phytochemicals have also been obtained in various food models as well (Honório et al., 2015).

Of the well-known aromatic and medicinal plants, mints of the *Lamiaceae* family include more than 20 species and natural hybrids. Their properties were discovered in ancient times, and today, several mint species and their EOs are exploited at various fields in medicine (Pagonopoulou et al., 2012; Koutroumanidou et al., 2013; Baser and Buchbauer, 2015). Their properties are related to the volatile compounds that constitute their EO, of which pulegone, carvone, and menthol have been extensively studied (Sivropoulou et al., 1995; Tassou et al., 1995, 2000; Tsai et al., 2013).

Piperitenone and its epoxide (PEO) and peroxide (PPO) derivatives are among the not so well-studied components occurring in EOs of various plants. These p-menthane type monoterpenes are natural constituents of the chemotypes of various plant species as *Calamintha nepeta* and *Calamintha glandulosa* (Kokkalou and Stefanou, 1990; Cook et al., 2007), *Satureja parvifolia* (Zygadlo et al., 1993), *Hyptis capitata* (Thoppil and Jose, 1995), *Tagetes patula* (Tamut et al., 2017), *Rosmarinus officinalis* (Gachkar et al., 2007), *Eucalyptus olida*, *Eucalyptus dives* (Gilles et al., 2010), and *Micromeria congesta* (Herken et al., 2012). However, it is in the *Lamiaceae* family and particularly in mint genus where PPO and PEO are among the main monoterpene components. Geographical origin, cultivation techniques, and even isolation methods are among the factors that influence plants’ EOs composition (Ciobanu et al., 2002; Teles et al., 2013) resulting most often in PPO and PEO being isolated from various *Mentha* spp. as minor ingredients and in percentages close to 1% (Duarte et al., 2005; Soković et al., 2009). In other mint species, however, these compounds are abundant and consisting a high percentage (from 40 to 85.4%) of their volatiles (Tomei et al., 2003; Esmaeili et al., 2006; Gachkar et al., 2007; Benayad et al., 2012). *Mentha* spp. rich in PPO/PEO have been reported from China (Zhao et al., 2013), Israel (Segev et al., 2012), Jordan (Abu-Al-Futuh et al., 2000), Lithuania (Venskutonis, 1996), and Greece (Kokkalou and Stefanou, 1990).

Epoxides, in general, are active compounds, and various studies have shown that, in mammals, they are able to react with nucleophilic groups in proteins (Guengerich, 2003) or acting as haptens, eliciting significant reactions to the skin (Nilsson et al., 2005). Sousa et al. (2009) reported an antinociceptive activity of PPO in mice and suggest that this effect is probably an indirect anti-inflammatory reaction.

Despite the fact that there are numerous reports concerning the antimicrobial activities of EOs against pathogens (Lang and Buchbauer, 2012), there are relatively limited studies exploiting the combined action of EOs and antibiotics (Langeveld et al., 2014; Owen and Laird, 2018). Among them, there is not, to our knowledge, a similar study concerning the combined action of PEO and antibiotics against clinical pathogens. Therefore, the aim of the present work was to assess the antimicrobial efficiency of PEO in combination with various antibiotics against *S. aureus* and *Escherichia coli* strains isolated from clinical samples.

## Materials and Methods

### Bacterial Strains

Twenty-eight clinical strains of *S. aureus* and 10 clinical strains of *E. coli* were used in the study. These strains were donated over time from “Metaxa” Anticancer Hospital and are now part of the frozen strain collection of the Laboratory of Microbiology, Biotechnology and Hygiene. Strains have been identified via VITEK 2 Compact (BioMerieux, Marcy-l’Étoile, France) and kept frozen in Tryptone Soya Broth (HiMedia Laboratories Pvt. Ltd., Mumbai, India) enriched with 30% glycerol until use. *S. aureus* strains were non-methicillin-resistant, and *E. coli* were non-extended spectrum β-lactamase producers as revealed by latex agglutination test (Oxoid^TM^ Ltd., United Kingdom) and Clinical and Laboratory Standard Institute (CLSI) double disk method (Gupta et al., 2013). *Staphylococcus aureus* ATCC^®^ 25923^TM^ and *E. coli* ATCC^®^ 25922^TM^ were used as reference strains. Before assays, all strains were incubated in the appropriate conditions to ensure optimal growth and purity. In the present study, no human or animal subjects were involved or any recorded data are used or maintained, and therefore, no ethics approval is required.

### Isolation and Characterization of Piperitenone Epoxide

Isolation and structural characterization of PEO followed the procedure as we previously described (Kimbaris et al., 2017). Briefly, aerial parts of full flowered plants of *M. spicata* were collected during July of 2018 from a wild-growing population in Sparti (South of Greece, Peloponisos). Plant material was air dried and cut into small pieces, and 500 g was subjected to hydrodistillation for 3 h, using a Clevenger type apparatus. The collected EO (1.8 ml/100 g dry wt) was dried over anhydrous magnesium sulfate, filtrated, and finally stored in sterile screw capped dark bottles at −22°C until use. Gas chromatographic–mass spectroscopic analysis (GC-MS) revealed, as expected, piperitone epoxide (23.2%) and PEO (50.9%) as the major ingredients. Part of the extracted crude EO (4 g) was fractioned by column chromatography on silica gel and eluted with a gradient of solvents of increasing polarity (pentane+diethyl). The resulted yellowish oil, identified as (+)-PEO (1.6 g, mixture of *cis* and *trans* diastereoisomers), was found in high purity (∼99.0%) according to GC-MS analysis. Structural determination was carried out by GC-MS and ^1^H- and ^13^C-NMR analysis, and the results were in agreement to previously reported (Kimbaris et al., 2017).

### Determination of Minimum Inhibitory Concentration

The minimum inhibitory concentration (MIC) of the various antibiotics was determined using commercially available 96-well microplate panels (Sensititre^®^, Trek Diagnostic System), preloaded with antibiotics (Table 1) following the method recommended by Clinical and Laboratory Standards Institute [CLSI] (2012). Assays were performed in Muller–Hinton broth (MHB) (HiMedia Laboratories Pvt. Ltd., Mumbai, India). In each well, 100 μl of MHB was added along with 20 μl of a diluted bacterial suspension in NaCl 0.85% to give a final concentration of 5 × 10^5^ CFU/ml. Wells without bacteria were used as negative controls. Plates were incubated for 16–24 h at 37°C, and growth was assessed after addition of tetrazolium dye [3-(4,5-dimethylthiazol-2-yl)-2,5-diphenyl tetrazolium bromide or MTT] (Sigma-Ardrich^®^) and further incubated for 60 min. The MIC was defined as the lowest antibiotic concentration without visible growth. Three independent assays were performed.

**TABLE 1:** Concentrations of antibiotics used for *Staphylococcus aureus* and *Escherichia coli* in the assay.

**Antibiotic**	***S. aureus* (test range in μg/ml)**	**Dilutions^∗^**	***E. coli* (test range in μg/ml)**	**Dilutions^∗^**
Amikacin	nt	–	64–0.5	7
Ampicillin	16–0.06	8	32–0.5	6
Cefepime	nt	–	32–0.5	6
Ceftazidime	nt	–	32–8	2
Ceftriaxone	64–0.03	11	64–0.06	10
Levofloxacin	32–0.06	9	8–0.008	10
Linezolid	8–0.5	4	nt	–
Meropenem	16–0.12	7	16–0.06	8
Minocycline	8–0.25	6	16–0.5	5
Penicillin	8–0.06	7	nt	–
Tigecycline	16–0.008	11	16–0.008	11
Vancomycin	32–0.12	8	nt	–

*^∗^Number of twofold dilutions starting from the largest concentration, nt, not tested.*

The MIC of PEO against pathogens was determined also by the broth microdilution method. A bacterial suspension was prepared in MHB from fresh overnight stock culture bearing a final concentration of 1.5 × 10^8^ CFU/ml or 0.5 McFarland turbidity units estimated using a dedicated densitometer (DensiCHEK^TM^ Plus, Biomerieux). In each well, 100 μl of MHB was added supplemented with dimethyl sulfoxide at a final concentration of 2% (*v*/*v*) to ensure oil solubility. In the first column of wells, 50 μl of PEO was added (1,024 μg/ml) and serially diluted horizontally to a final concentration of 0.25 μg/ml. An aliquot of 50 μl from bacterial suspension was added to each well. Plates were covered and incubated at 37°C for 16–24 h. The MIC was defined as the lowest concentration with no visible growth.

### Checkerboard Assay

To study the combined action of antibiotics and PEO, five Sensititre^®^ plates were used for every strain with each one of the plates containing a different concentration of PEO. The concentrations of PEO used were selected on the basis of MIC values previously determined (four twofold dilutions starting at 32 mg/l (i.e., 32, 16, 8, and 4 μg/ml). Higher concentrations of PEO could be effective also but unworthy for clinical exploitation. MHB used in combined experiments was supplemented with dimethyl sulfoxide (Sigma-Ardrich^®^) at a final concentration of 2% (*v*/*v*). Incubation conditions and interpretation of results were similar to the ones already described. The combined action of the antibiotics and PEO was expressed in terms of fractional inhibitory concentration (FIC) index (FICI) equal to the sum of FICs for each drug. The FIC is defined as the MIC of each substance or drug used in combination divided by the MIC of the substance or drug used alone based on the following equation (Doern, 2014):

(1) FICI=FIC+PEOFIC=Drug(MIC⁢inPEO⁢combination/MIC⁢alonePEO)+(MIC⁢inDrug⁢combination/MIC⁢aloneDrug)

The results were considered as a synergistic effect if the FICI of the combination is ≤0.5, additive when 0.5 < FICI < 1, indifferent when 1 < FICI ≤ 2, and antagonistic for FICI > 2 (EUCAST, 2000). All experiments were performed in triplicate.

### Statistical Analysis

Comparison of the mean MIC values of PEO and antibiotics against *S. aureus* and *E. coli* was performed with the Mann–Whitney non-parametric procedure at an alpha level of 0.05.

## Results

### Minimum Inhibitory Concentration of PEO and Antibiotics

In our study, overall MIC values of PEO ranged between 32 and 1,024 μg/ml (Table 2). The average MIC (μg/ml) of PEO against the 10 *E. coli* strains was 512.2 ± 364.7 μg/ml, which is significantly higher (Mann–Whitney *p* < 0.05) than 172.8 ± 180.7 μg/ml observed for the 28 *S. aureus* strains.

**TABLE 2:** Minimum inhibitory concentrations (μg/ml) of piperitenone epoxide (PEO) and antibiotics against clinical isolates of *Escherichia coli* and *Staphylococcus aureus*.

**Minimum inhibitory concentration (μg/ml)^∗^**													
**Strain**	**PEO**	**AMK**	**AMP**	**FEP**	**CAZ**	**CRO**	**LVX**	**LZD**	**MEM**	**MIN**	**PEN**	**TGC**	**VAN**
*E. coli 1*	256	4	32	16	16	2	0.06	nt	2	2	nt	0.5	nt
*E. coli 2*	512	4	32	4	32	4	0.12	nt	1	4	nt	0.5	nt
*E. coli 3*	1024	1	8	4	32	4	2	nt	2	16	nt	0.25	nt
*E. coli 4*	512	4	32	16	32	8	0.06	nt	2	2	nt	0.25	nt
*E. coli 5*	512	4	32	32	16	64	2	nt	4	16	nt	0.12	nt
*E. coli 6*	128	4	32	2	32	32	8	nt	2	16	nt	0.12	nt
*E. coli 7*	128	4	32	8	16	64	8	nt	4	16	nt	0.12	nt
*E. coli 8*	512	4	32	16	32	8	0.06	nt	2	2	nt	0.25	nt
*E. coli 9*	1024	1	8	4	32	4	2	nt	2	16	nt	0.25	nt
*E. coli 10*	512	4	8	2	32	8	0.06	nt	2	2	nt	0.25	nt
*E. coli 25922*	512	1	4	<0.5	<8	0.05	0.06	nt	0.06	2	nt	0.25	nt
*S. aureus 1*	256	nt	32	nt	nt	8	0.5	4	0.5	0.25	0.5	0.12	2
*S. aureus 2*	64	nt	32	nt	nt	16	0.5	16	2	2	1	0.12	2
*S. aureus 3*	128	nt	0.25	nt	nt	4	0.5	8	0.25	1	0.25	0.06	1
*S. aureus 4*	128	nt	2	nt	nt	4	0.25	8	0.5	0.5	0.25	0.25	1
*S. aureus 5*	64	nt	32	nt	nt	16	32	2	1	0.25	0.5	0.06	1
*S. aureus 6*	128	nt	16	nt	nt	8	4	8	0.25	0.25	0.5	0.12	1
*S. aureus 7*	128	nt	16	nt	nt	16	0.25	4	0.12	0.25	0.25	0.06	1
*S. aureus 8*	256	nt	0.12	nt	nt	4	0.5	4	0.5	0.5	0.06	0.12	2
*S. aureus 9*	128	nt	16	nt	nt	8	0.25	8	0.5	0.5	0.5	0.12	1
*S. aureus 10*	64	nt	16	nt	nt	4	1	4	0.5	0.5	0.5	0.25	1
*S. aureus 11*	256	nt	16	nt	nt	16	8	8	2	1	1	0.12	1
*S. aureus 12*	128	nt	16	nt	nt	8	0.5	8	0.25	1	0.5	0.12	2
*S. aureus 13*	128	nt	16	nt	nt	8	1	8	0.5	1	1	0.12	2
*S. aureus 14*	64	nt	16	nt	nt	4	0.5	8	0.25	0.5	0.06	0.12	1
*S. aureus 15*	256	nt	16	nt	nt	8	1	4	0.25	0.5	0.12	0.12	1
*S. aureus 16*	256	nt	16	nt	nt	4	0.5	8	0.12	1	0.5	0.25	1
*S. aureus 17*	256	nt	16	nt	nt	4	1	8	0.25	1	0.5	0.06	1
*S. aureus 18*	32	nt	16	nt	nt	16	2	8	0.25	8	0.06	0.25	1
*S. aureus 19*	256	nt	16	nt	nt	8	2	8	0.25	1	0.12	0.12	1
*S. aureus 20*	64	nt	16	nt	nt	8	1	8	0.5	2	0.5	0.25	1
*S. aureus 21*	32	nt	16	nt	nt	8	0.5	8	0.25	8	0.5	0.12	1
*S. aureus 22*	128	nt	16	nt	nt	16	4	8	0.25	8	0.5	0.25	2
*S. aureus 23*	32	nt	0.25	nt	nt	4	0.5	8	0.25	1	0.25	0.06	4
*S. aureus 24*	32	nt	16	nt	nt	8	0.5	8	0.25	1	0.12	0.12	2
*S. aureus 25*	1024	nt	16	nt	nt	16	0.12	4	0.12	0.25	0.12	0.06	2
*S. aureus 26*	256	nt	0.12	nt	nt	4	0.5	16	0.25	0.5	0.06	0.12	2
*S. aureus 27*	128	nt	16	nt	nt	8	1	4	0.25	0.5	0.5	0.12	1
*S. aureus 28*	128	nt	16	nt	nt	4	1	8	0.25	0.5	0.12	0.25	1
*S. aureus 25923*	64	nt	0.25	nt	nt	4	0.06	0.5	0.12	0.25	0.12	0.06	0.25

*^∗^Mean values of three replicates, nt, not tested. Piperitenone epoxide (PEO), Amikacin (AMK), Ampicillin (AMP), Cefepime (FEP), Ceftazidime (CAZ), Ceftriaxone (CRO), Levofloxacin (LVX), Linezolid (LZD), Meropenem (MEM), Minocycline (MIN), Penicillin (PEN), Tigecycline (TGC), Vancomycin (VAN).*

Based on the ecological cutoff values presented by EUCAST (2019), *E. coli* strains were proven to be multiresistant in almost all antibiotics. All 10 strains were resistant to cefepime (FEP), ceftazidime (CAZ), ceftriaxone (CRO), and meropenem (MEM) and sensitive to amikacin (AMK) and tigecycline (TGC). The resistance percentage of *E. coli* isolates to the rest of antibiotics ranged from 50 to 70%.

Similarly, an increased resistance to several antibiotics was observed for *S. aureus* isolates particularly in ampicillin (82.1%), linezolid (71.4%), and minocycline (82.1%). Resistance ranged from 10 to 25% for the rest of the antibiotics, while none of the *S. aureus* isolates was resistant to tigecycline and only one to vancomycin (3.6%). Resistance to penicillin (>1 μg/ml) was recorded in three strains. *E. coli* and *S. aureus* reference strains were constantly exhibiting sensitivity to the majority of the drugs.

### Fractional Inhibitory Concentration of PEO and Antibiotics

**FIGURE 1:**
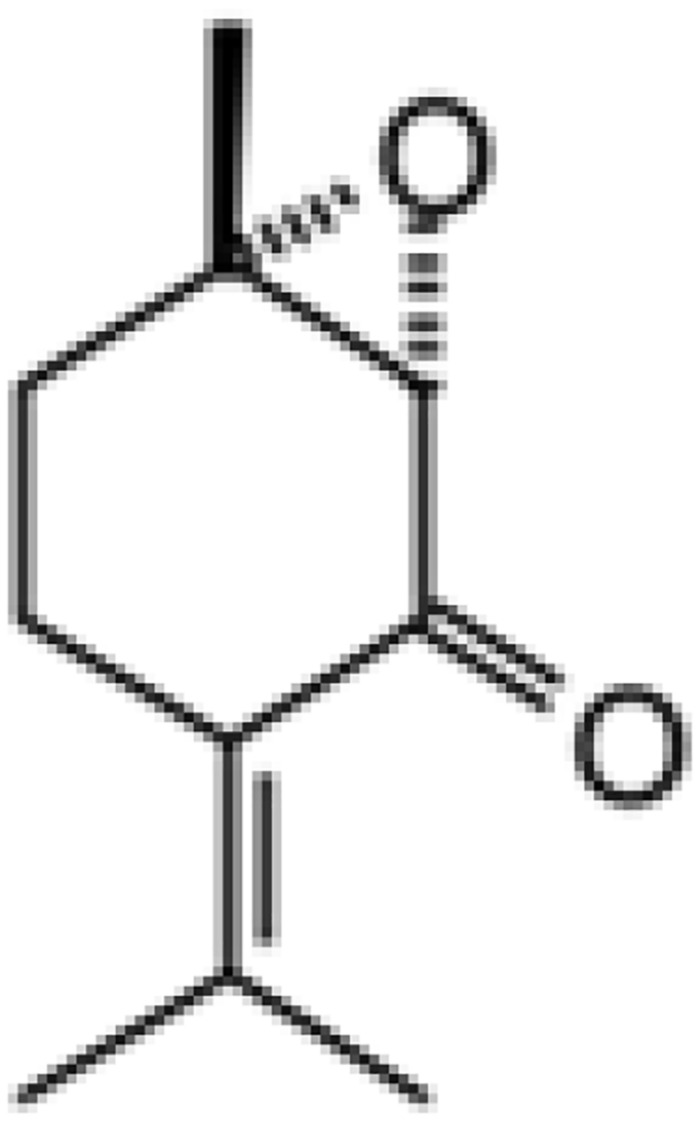
Chemical structure of piperitenone epoxide (PEO).

In our results, out of the 90 assays (240 with the replications) to determine the FICs from the combination of antibiotics and PEO on clinical isolates of *E. coli*, synergy, according to EUCAST (2000), was detected in 73 or 81.1% (Table 3). A complete synergistic effect was recorded in the case of amikacin/PEO, ampicillin/PEO, ceftazidime/PEO, meropenem/PEO, and minocycline/PEO, whereas 50–80% synergism was observed for the rest of the antibiotics when combined with PEO. Additive effects were exhibited by 10% of the FICIs and indifferent by 5.5%. No antagonistic interactions were recorded during the *E. coli* experiments (Figure 1).

**TABLE 3:** Mean values and interpretations according to EUCAST (2000) of fractional inhibitory concentration indexes (FICI) for piperitenone epoxide (PEO) and antibiotics against 10 *Escherichia coli* strains.

**Strain**	**AMK**	**AMP**	**FEP**	**CAZ**	**CRO**	**LVX**	**MEM**	**MIN**	**TGC**
*E. coli 1*	0.50	0.50	0.50	0.50	0.63	1.50	0.38	0.38	0.49
*E. coli 2*	0.38	0.50	0.31	0.50	0.31	0.56	0.37	0.19	0.49
*E. coli 3*	1.50	0.16	0.38	0.19	0.50	0.16	0.19	0.38	1.06
*E. coli 4*	0.50	0.38	0.38	0.31	0.19	1.06	0.38	0.38	0.30
*E. coli 5*	0.75	0.50	0.38	0.50	0.13	0.28	0.13	0.50	0.56
*E. coli 6*	0.50	0.38	0.75	0.38	0.50	0.50	0.38	0.50	1.50
*E. coli 7*	1.50	0.31	0.50	0.50	0.56	0.26	0.31	0.50	0.75
*E. coli 8*	0.50	0.31	0.31	0.31	0.38	0.13	0.25	0.50	0.37
*E. coli 9*	1.13	0.38	0.31	0.31	0.50	0.53	0.25	0.38	0.30
*E. coli 10*	0.50	0.19	0.56	0.38	0.38	1.06	0.31	0.19	0.30
*E. coli 25922*	0.50	0.16	0.31	0.19	0.19	0.19	0.31	0.19	0.37
^∗^Synergy (%)	60	100	80	100	80	50	100	100	60
^∗^Additive (%)	10	0	20	0	20	20	0	0	20
^∗^Indifference (%)	30	0	0	0	0	30	0	0	20
^∗^Antagonism (%)	0	0	0	0	0	0	0	0	0

*^∗^Reference strain not included. Amikacin (AMK), Ampicillin (AMP), Cefepime (FEP), Ceftazidime (CAZ), Ceftriaxone (CRO), Levofloxacin (LVX), Meropenem (MEM), Minocycline (MIN), Tigecycline (TGC).*

**FIGURE 2:**
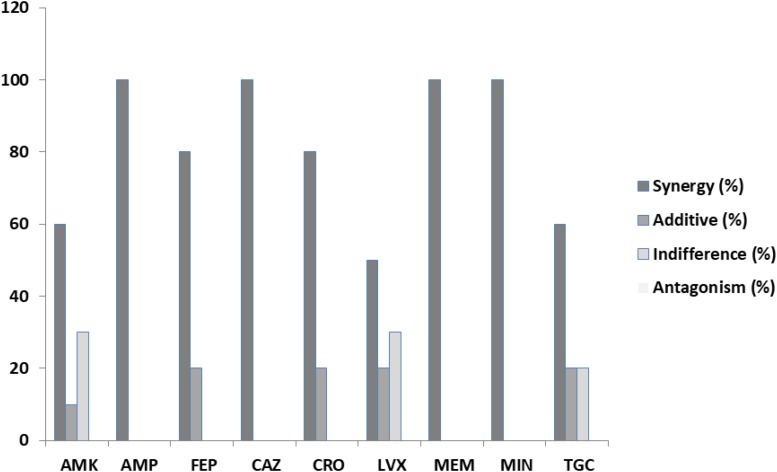
Percentages of synergy, additive, indifference, and antagonistic interactions between nine commercial antibiotics and piperitenone epoxide against clinical isolates of *Escherichia coli* from checkerboard assays.

**FIGURE 3:**
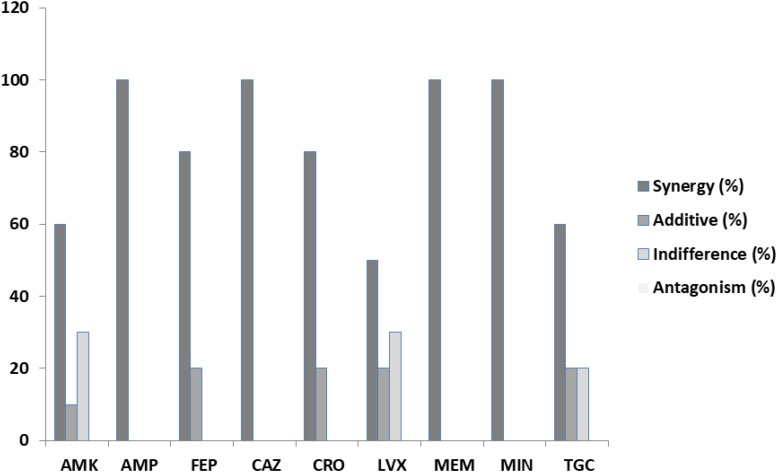
Percentages of synergy, additive, indifference, and antagonistic interactions between nine commercial antibiotics and piperitenone epoxide against clinical isolates of *Staphylococcus aureus* from checkerboard assays.

Among the *S. aureus* assays (Table 4), synergism was observed for all antibiotics but in variable percentages. Specifically, 67.86% of the ceftriaxone/PEO, levofloxacin/PEO, and linezolid/PEO combinations exhibited synergism (FICI ≤ 0.5). Similarly, synergism was observed in 60.71% of the vancomycin/PEO combination, 57.14% of ampicillin/PEO, 53.57% of penicillin/PEO, 42.86% minocycline/PEO, 35.71% of meropenem/PEO, and finally 3.57% of tigecycline/PEO. The frequency of additive effects ranged between 0 and 21.43%. Indifference was recorded in all combinations of antibiotics/PEO (ranged from 10.71 to 67.86% per antibiotic), and finally, antagonistic effects were recorded in six out the nine combinations of antibiotics/PEO and in ranges from 10.71% and up to 25% of the assays (Figures 2, 3).

**TABLE 4:** Mean values of fractional inhibitory concentration indexes (FICI) for piperitenone epoxide and antibiotics against 28 *Staphylococcus aureus* strains.

**Strain**	**AMP**	**CRO**	**LVX**	**LZD**	**MEM**	**MIN**	**PEN**	**TGC**	**VAN**
*S. aureus 1*	0.31	0.25	0.16	0.38	3.00	1.50	0.50	0.63	0.38
*S. aureus 2*	0.56	0.63	0.51	0.56	2.50	0.63	0.56	2.00	0.75
*S. aureus 3*	0.73	0.50	0.49	0.50	1.13	0.37	3.00	2.00	0.50
*S. aureus 4*	0.38	0.50	0.12	0.50	0.49	0.49	0.31	2.00	1.50
*S. aureus 5*	0.51	0.56	0.52	1.00	0.37	3.00	0.63	3.00	1.25
*S. aureus 6*	0.38	0.50	0.27	0.38	0.25	0.37	0.50	2.00	0.50
*S. aureus 7*	0.38	0.31	0.49	0.50	3.00	1.25	3.00	2.00	0.50
*S. aureus 8*	1.25	0.38	1.13	0.50	0.37	1.13	1.25	2.00	0.63
*S. aureus 9*	0.38	2.00	0.49	0.38	0.49	0.49	0.50	1.50	0.50
*S. aureus 10*	0.51	0.75	0.51	0.75	1.13	0.49	2.50	2.00	0.75
*S. aureus 11*	0.50	0.38	0.50	0.38	0.50	1.13	0.25	1.25	0.38
*S. aureus 12*	0.26	0.38	0.37	0.38	1.48	0.50	0.38	3.08	0.38
*S. aureus 13*	0.38	0.38	0.31	0.50	2.50	1.25	0.50	2.58	0.38
*S. aureus 14*	0.56	1.50	0.31	0.63	1.48	0.56	2.50	2.00	0.75
*S. aureus 15*	0.38	0.50	0.31	0.50	0.25	0.37	0.38	2.33	0.38
*S. aureus 16*	0.38	0.38	0.25	0.38	1.25	0.63	0.38	2.00	0.38
*S. aureus 17*	0.25	0.50	0.19	0.50	1.13	1.13	0.38	1.25	0.38
*S. aureus 18*	3.00	0.56	1.02	1.25	3.00	1.25	4.50	2.00	0.75
*S. aureus 19*	0.38	0.25	0.28	0.38	0.37	0.38	0.50	1.50	0.38
*S. aureus 20*	3.00	0.38	0.51	0.63	1.24	1.50	0.75	2.00	0.75
*S. aureus 21*	1.25	1.13	3.00	1.13	1.48	1.25	1.13	2.00	1.50
*S. aureus 22*	0.38	0.31	0.50	0.38	0.31	1.25	0.50	1.50	0.38
*S. aureus 23*	3.00	1.25	3.00	1.25	2.48	3.00	9.00	5.17	1.25
*S. aureus 24*	1.13	1.25	3.00	1.13	1.13	2.25	2.13	2.00	2.00
*S. aureus 25*	0.28	0.19	0.28	0.38	1.13	0.15	0.50	1.13	0.31
*S. aureus 26*	1.13	0.38	0.25	0.38	5.00	0.38	1.13	0.63	0.38
*S. aureus 27*	0.50	0.50	0.31	0.50	1.48	0.50	0.38	0.75	0.50
*S. aureus 28*	0.38	0.50	0.31	0.38	0.31	0.50	0.50	0.49	0.50
*S. aureus 25923*	0.28	0.25	0.16	0.38	0.25	0.37	0.50	0.63	0.38
^∗^Synergy(%)	57.14	67.86	67.86	67.86	35.71	42.86	53.57	3.57	60.71
^∗^Additive(%)	17.86	14.29	14.29	17.86	0	10.71	10.71	10.71	21.43
^∗^Indifference(%)	14.29	17.86	7.14	14.29	39.29	35.71	10.71	67.86	17.86
^∗^Antagonism (%)	10.71	0	10.71	0	25	10.71	25	17.86	0

*^∗^Reference strain not included. Ampicillin (AMP), Ceftriaxone (CRO), Levofloxacin (LVX), Linezolid (LZD), Meropenem (MEM), Minocycline (MIN), Penicillin (PEN), Tigecycline (TGC), Vancomycin (VAN).*

## Discussion

Recent data suggest that the estimated burden of infection with antibiotic-resistant bacteria is substantial and has increased over the last years forcing toward comprehensive European and global action plans (Cassini et al., 2019). Based on this increase in antibiotic resistance, products of natural origin as the EOs alone or in combination with other agents could be a promising alternative (Lang and Buchbauer, 2012; Langeveld et al., 2014). EOs are secondary metabolites of aromatic and medicinal plants and play an important role in their proliferation and defense (Baser and Buchbauer, 2015). Most of them are terpene and terpenoid mixtures with a lipophilic nature. However, since EOs are complex mixtures with variable and unsteady composition (Thoppil and Jose, 1995; Teles et al., 2013), they are hardly useful for medicinal use (Owen and Laird, 2018).

Piperitenone and piperitone are among the main components of various EOs isolated from aromatic and medicinal plants, particularly from *Mentha* sp., which, according to Mahboubi and Haghi (2008), belongs to piperitone/piperitenone type of EOs. From the limited available literature, it is known that both compounds have proven antimicrobial activities against *Mucor rouxii* (Bakkali et al., 2008), *S. aureus* (Mahboubi and Haghi, 2008), and *Aspergillus flavus* (Cárdenas-Ortega et al., 2005). Early studies have shown that PEO increased the antimicrobial activity of furazolidone and nitrofurantoin (Shahverdi et al., 2004). Their mode of action is mostly associated with their lipophilic nature, the accumulation in membranes, and the sensitization of the phospholipidic bi-layer of the cell membrane causing an increase in permeability and leakage of various vital constituents (Conner, 1993; Moreira et al., 2005). However, both epoxides have not been investigated for their synergism in combination with antibiotics.

The European Committee for Antimicrobial Susceptibility Testing (EUCAST) has proposed (EUCAST, 2000) a classification of the FICI occurring from a combination of antibiotics according to which any FICI ≤ 0.5 denotes synergy, additive when 0.5 < FICI ≤ 1, indifference when 1 < FICI < 2, and antagonism when FICI ≥ 2. Another interpretation for checkerboard assays was proposed by Odds (2003), arguing about the reproducibility problems arising from the use of this methodology in comparison to others less prone to errors (Lewis et al., 2002) but also less popular to microbiologists. According to Odds (2003), a synergy could be defined if FICI was ≤0.5 and antagonism when FICI > 4. For FICI values between 0.5 and 4, “no interaction” should be stated. According to that author, such a conservative interpretation would be helpful for comparison purposes of the data published in the antimicrobial field. To deal with the reproducibility problems of the multiple checkerboard assays for the estimation of MIC and FIC, Fratini et al. (2017) proposed a modification in which both MIC and FIC are estimated in the same microplate. According to those authors, similar errors occur for the two estimations, and therefore, a synergistic effect is detected when FICI value < 1, a cumulative effect when FICI value = 1, an indifferent effect when 1 < FICI ≤ 2, and an antagonistic effect when FICI value > 2 (Fratini et al., 2017). In *E. coli* experiments which exhibited synergism, the mean reduction in the effective drug doses were 4-fold for amikacin, ceftazidime, minocycline and tigecycline, 8-fold for ampicillin and ceftriaxone, 16-fold for cefepime and meropenem, and up to 133-fold reduction for levofloxacin. The corresponding mean reductions for the drugs assayed in *S. aureus* strains were 8-fold for ampicillin, ceftriaxone, meropenem, and penicillin; 4-fold for linezolid, minocycline, tigecycline, and vancomycin; while there was a 66-fold mean reduction in the effective dose for levofloxacin. It is clear from our data that the use of PEO in combination with the various drugs gave some positive results about the synergistic effects, and despite the initial and relatively high MIC values of this compound, it finally reduced considerably the effective doses of the drugs even in the case of *E. coli* which, as a Gram(−) microorganism, is more resistant to the EOs antibacterial action (Lang and Buchbauer, 2012).

The mechanism of action of PEO is not known, and this is also the case for any EO or their component. However, we can speculate on the site of action since there are relevant scientific evidence about molecules highly analogous to the PEO like carvacrol, thymol, and *p*-cymene. Among others, Ultee et al. (2002), Gill and Holley (2006), and Di Pasqua et al. (2007) have studied the way carvacrol, a key compound in oregano EO, acts on the bacterial cell. They proposed that the membrane disruption and destabilization leading to leakage of cell ions, fluidization of membrane lipids, and the reduction in the proton motive force is the primary target for those molecules. Additional modes of action, following the disruption of the membrane with potential intracellular targets as the inhibition of ATPase activity, has been also proposed for thymol and *p*-cymene (Lambert et al., 2001; Ultee et al., 2002; Trombetta et al., 2005; Di Pasqua et al., 2010). In a similar way, we can speculate that the destabilization of the membrane by PEO results in a more efficient diffusion of drugs in the membrane or in the cell, thus exhibiting a higher activity being at a lower dose. However, in our study, decisive data like the minimum bactericidal concentration or time of kill were not estimated, and no molecular approaches were involved; therefore, no specific assumptions about the bactericidal effects can be drawn (Wagner and Ulrich-Merzenich, 2009). Consequently, it is apparent that more research is needed to demonstrate the feasibility of use for the combined action of drugs and EOs constituents.

## Conclusion

The multidrug-resistant microorganisms represent an increasingly widespread hazard. Essential oils could be an effective alternative to drugs in human and veterinary medicine. Particularly, various EOs compounds as the PEO, due to their promising synergistic or additive action, could be employed to reduce the effective drug dose against common pathogens, thus limiting the overuse of antibiotics or reducing their resistance.

## Data Availability Statement

The datasets generated for this study are available on request to the corresponding author.

## Author Contributions

AK, SP, and IM were responsible for the isolation and chemical characterization of PEO from EOs. CT, OP, and ES concerned with susceptibility testing. CV, MF, and CB performed the checkerboard assays. VP was responsible for the strains. AA and EB conceived the study and actively involved in all of the above.

## Conflict of Interest

The authors declare that the research was conducted in the absence of any commercial or financial relationships that could be construed as a potential conflict of interest.
